# Climate Change and Health: Local Government Capacity for Health Protection in Australia

**DOI:** 10.3390/ijerph20031750

**Published:** 2023-01-18

**Authors:** James C. Smith, Harriet Whiley, Kirstin E. Ross

**Affiliations:** College of Science and Engineering, Flinders University, Adelaide 5042, Australia

**Keywords:** climate change, local government, environmental health, environmental health officers, EHOs

## Abstract

Climate change is the greatest global health threat of the 21st century, with numerous direct and indirect human health consequences. Local governments play a critical role in communities’ response to climate change, both through strategies to reduce emissions and adaption plans to respond to changing climate and extreme weather events. Australian local government environmental health officers (EHOs) have the relevant skills and expertise to inform and develop adaptation plans for health protection in the context of climate change. This study used an online survey followed by phone interviews of local government management to determine the extent to which EHOs are involved in adaptation planning in health protection climate change plans. Questions were also asked to determine whether local councils are aware of EHOs’ capability to contribute and to gauge the willingness of management to provide EHOs with the workload capacity to do so. The findings demonstrated that although climate adaptation and mitigation planning is occurring in local government, it is not including or considering the public health impacts on the community. Primarily, it was found that this oversight was due to a lack of awareness of the health impacts of climate change outside of a disaster or emergency scenario. Currently, EHOs are an untapped source of knowledge and skills that can contribute to climate change adaption planning. To support this, a framework of local environmental health practice was developed to assist the reconceptualization of the scope of practice required for the planning and response to climate change.

## 1. Introduction

The 2015 report of the Rockefeller Foundation–Lancet Commission on Planetary Health states that human health is under threat from far-reaching changes to the structure and function of the Earth’s natural systems. The health effects from ‘… climatic change, ocean acidification, land degradation, water scarcity, overexploitation of fisheries, and biodiversity loss pose serious challenges to the global health gains of the past several decades…” [1]. The World Health Organization [2] has stated that climate change is the greatest health challenge of the 21st century and threatens to undermine over half a century of global health improvements by destabilizing the social and environmental determinants of health, including people’s access to clean air, safe drinking water, sufficient food, and secure shelter. There are also concerns about the spread of climate-sensitive infectious diseases, vector-borne diseases, water- and food-borne illnesses, population displacement, and reduced access to health services [2,3,4,5]. In Australia, the threat of climate change is even more pertinent as the health of Australians is highly vulnerable to its climate and weather extremes [6]. This is recognized by the Environmental Health Standing Committee (enHealth) of the Australian Health Protection Principal Committee 2020–2023 strategic plan [7] which identifies climate change as a strategic priority. 

The reality is that climate change is affecting the health and wellbeing of communities. A strong local capacity is needed to ensure that there is clean water, safe food, housing, and sanitation, and to communicate risks and respond to emergencies. This provides the foundation for adaptation and resilience [5]. Sheehan et al. [4] observed that local government is the first line of defence and the backbone of public health preparedness, surveillance, and response. Local government is uniquely positioned in this role through its intimate knowledge of its population needs. Austin et al. [8] also noted that local public health authorities are well positioned to reduce the health burden of climate change due to their implementation role, and their proximity to climate change impacts. The Australian government, through its National Climate Resilience and Adaptation Strategy 2021–2025, stated that local government has an essential role to play in ensuring that local circumstances are adequately considered in the overall adaptation responses to climate change [9]. State local government associations that represent the interests of local councils and their communities have also acknowledged that local councils have a key role in adaptation to climate change and that these peak bodies, in turn, have a clear role in assisting their member councils to do so [10]. This assistance has taken the form of general advice or specific initiatives, such as developing a formal action plan [11], a climate risk management framework [12], a workshop package to develop a climate change action plan [13], or a Climate Change Action Framework [14].

‘Adaptation’ is defined by the Intergovernmental Panel on Climate Change (IPCC) as the process of adjustment to actual, or expected climate change and its effects, to moderate harm or exploit beneficial opportunities. ‘Adaptive capacity’ refers to the available resources that can be used ‘… to prepare for and undertake actions to reduce adverse impacts, moderate harm, or exploit beneficial opportunities’ [15]. As pointed out by Ebi and Semenza [16], the analogous term for ‘adaption’ in public health is ‘prevention’. Thus, adaptation can be those actions taken in advance of climate change impacts in response to emerging health risks. Prevention consists of three major strategies: health protection, disease prevention, and health promotion [17]. Environmental health officers (EHOs) are the only professionals that have historically provided, and currently provide, health protection services to the community at the local level [18]. Unfortunately, the majority of EHOs have been constrained in their roles to those statutory obligations imposed on local councils with few being utilized, as was intended by the New Environmental Health introduced in 1999, in the investigation and assessment of public health risk factors, and development of risk mitigation strategies, which are an integral part of local adaptation to climate change [19]. 

In Australia, EHOs are employed specifically by councils to implement the health protection statutory obligations imposed on councils and must undertake prescribed qualification studies at the under-, or postgraduate levels. As a result, they have professional scientific knowledge and skills in environmental health and risk assessment, and in the administration of relevant legislation [20,21,22,23]. Subsequently, they have relevant skills and knowledge which can be used in adaptation planning for health protection in the context of climate change. The aim of this research was to determine whether local councils are aware of the capacity of EHOs to contribute to the adaptation planning in health protection and, secondly, to gauge the willingness of council management to re-prioritize existing service tasks to provide EHOs with the time to contribute to this important area. To the authors’ knowledge, this is the first study of this kind undertaken anywhere in the world. 

## 2. Materials and Methods

An online survey was created using Qualtrics^®^. The survey was directed to the executive responsible for environmental health services at local councils designated as cities across Australia. City councils were selected as, generally, they are more diverse, challenging, and complex in terms of structure, function, and the interrelationships of issues [24]. Further, 72% of the total population resides in major cities [25], and it is expected that these populations will be especially affected by climate change [26]. 

In local governments, all executive functions rest with the chief executive officer (or equivalent), who is responsible for the administration of the organization [27,28]. Usually, this will require a management structure based on divisions [27] to integrate all activities across the organization into one corporate entity [29]. The executive team members responsible for these divisions have delegation from, and directly report to, the CEO. These executives are the focus of the survey. 

The survey aim was to investigate executives’ knowledge and perception of EHO-associated activities and their engagement/relevance to councils’ climate change preparedness and response. The survey was developed to determine whether the respondent was executive management, using two different questions, and then three simple questions were asked of the participant, with some suggested answers and free text boxes. The draft survey was trialled with two senior managers, one from a rural and one from a metropolitan council. The testing consisted of advising participants about the general nature of the research being undertaken, requesting participants to complete the surveys, and then obtaining their immediate feedback after each had completed the survey. This feedback informed the development of the final survey (Supplementary Figure S1).

Executives were recruited to participate in the survey across Australia using (where possible) individually directly addressed emails. When this was not possible, the emails were sent to the general council email. Emails asked the addressees whether they would be willing to participate and, if so, to access the survey via the QR code or URL provided. The survey was opened on 11 May 2022 and closed on 14 June 2022. 

The final question in the survey asked whether participants were willing to be interviewed further about their responses. Follow up interviews were conducted over the phone using the ‘prompt questions’ presented in Supplementary Figure S2. This study was approved by the Flinders University Human Social and Behavioural Research Committee, (approval number 4682).

## 3. Results

The online survey received 52 responses. This included 15 from NSW, 15 from Victoria, eleven from South Australia, six from Western Australia, three from Queensland, and two from Tasmania. Twenty-four of the responses were from officers below the executive level and included environmental health officers, environmental health team leaders, or service managers. Twenty-eight surveys were completed by executives, who were the focus of this study, and these responses were further integrated separately. The 28 responses from executives included six from NSW, eight from South Australia, seven from Victoria, four from Western Australia, two from Queensland and one from Tasmania. Figure S3 presents the number of responses from executives compared with population data for each state. This shows a slight underrepresentation for Queensland and a slight overrepresentation for South Australia. Of these, seven executives (two from Victoria, two from Western Australia, and one each from South Australia, Tasmania, and Queensland) participated in the follow up phone interviews. 

### 3.1. Executives’ Perception of the Local Government Environmental Health Role

**Figure 1:**
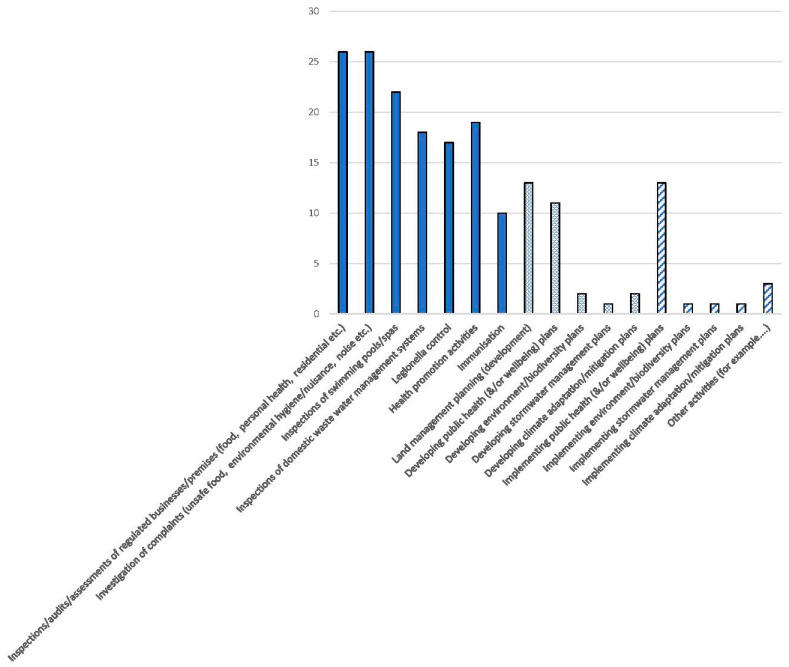
Main activities of EHOs identified by their executives (*n* = 28) (regulatory activities = solid fill, planning = cross-hatched, and implementation = hatched lines).

Figure 1 presents the survey results from the 28 executives to the question “What are the main activities of these EHOs? (tick all that apply).” This figure shows that the regulatory activities were more frequently identified by executives, compared with the activities related to planning and implementation. Survey participants were provided a free text box to include any additional activities. The responses included additional regulatory activities such as arborvirus control, asbestos, clandestine laboratories, recreational water monitoring, local laws and temporary events, accommodation parks, footpath dining, liquor licence applications, noise reports, assessment for development approvals, and public health complaints—green pools, hoarding, vermin, noise, and pollution. 

Of interest were the 24 responses from officers below the executive level, that is, EHOs, team leaders, and service managers, who identified emergency management planning, relief, and recovery as EHO activities that were not listed by executives. These officers also included activities relating to tobacco control, domestic squalor, assessment of building and planning developments, and solid fuel heaters.

### 3.2. Local Government Environmental Health and Climate Change

In response to the question “If EHOs are involved in climate change/mitigation planning, what specific roles do they fulfil?”, only four executives indicated that the EHOs were involved in climate change/mitigation planning, with specific roles being:

1. Project team member (1).
2. Plan development—provider of advice/information (1).
3. Plan implementation—responsible for designated tasks (0 chose this option).
4. Other (2 responses—specifically “Specialist advice only” and “Provision of information to food businesses/health premises, etc.”).

All team leaders or managers (L3 and L4) indicated that there were no EHOs involved in climate change/mitigation planning, with one comment “Not applicable as it is undertaken by a separate team”.

**Figure 2:**
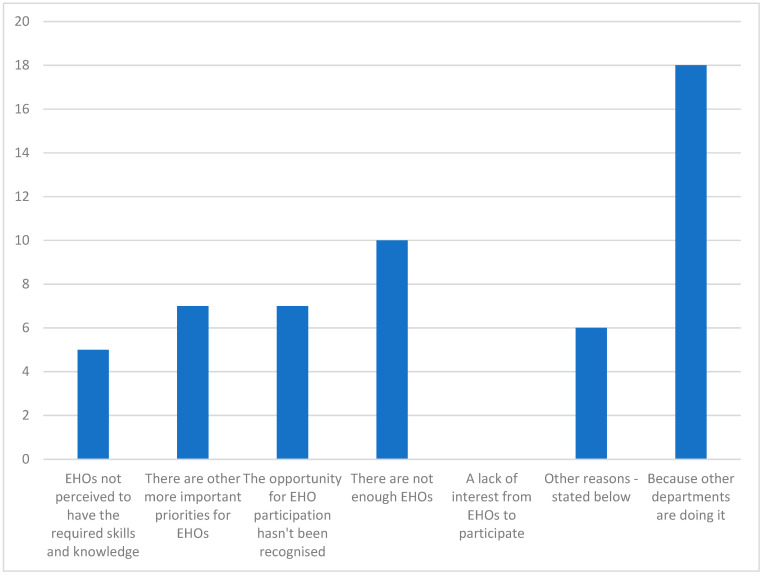
Reasons executives provided as to why local government environmental health officers (EHOs) were not being involved in climate change planning (*n* = 28).

In response to the question “If EHOs are not involved in climate change/mitigation planning and the health impacts associated with climate change, why?”, the most common response was that the lack of involvement was “Because other departments are doing it” (Figure 2). Interestingly, no one chose the prompt “A lack of interest from EHOs.” The other reasons identified by executives using the free text box provided with this question included:

1. No time to develop climate change adaptation/mitigation as a discrete task. It is built into any other relevant task such as the Stormwater Management Plan.
2. Currently not a major focus of the city.
3. Separate Environmental Services team in Sustainable Assets.
4. Lack of understanding of the role they can play.
5. We have a Sustainability Officer.
6. Climate change plans are managed by other environmental specialists within council.

Interestingly, the team leaders and managers (L3 and L4), again, provided slightly different responses than the executive staff and the gave the reasons below:

1. Considerable cost shifting to Council EHOs over the years and EHOs have several priorities they are juggling.
2. EHO provide advice to staff where their primary role is in climate change/mitigation planning.
3. We have a Natural Environment Program that has developed a carbon reduction strategy. EHOs work in a regulatory space, I’m not sure how we could make the jump to climate change planning without a regulatory framework in place.
4. As far as I know the city has not identified this as an area of priority.
5. Lack of capacity due to workload.

The follow up interviews were conducted with the seven executives who were willing to be interviewed. The follow up interview aimed to further investigate the issues arising from the responses and interrogate the response “other departments are doing it [climate change planning].” Responses from the interview participants were grouped according to climate change themes including sustainability, water, zero-carbon planning, adaptation, and resilience, and, specifically, health components of climate change (emergency management, heatwaves, flooding, and vector-borne disease).

Several interviewees referred to sustainability, water, zero-carbon planning, and other areas that are part of their planning teams but, almost exclusively, this did not include EHOs. The interviewees’ responses suggest minimal health input into the adaptation and resilience side of climate change planning. All executives indicated that they have little capacity for providing the health component of climate change beyond emergency management. When prompted, all executives indicated that health inputs were coming from EHOs in response to emergency management planning, particularly around heatwaves, flooding, and vector-borne disease. One respondent did note that there was more involvement [in planning] and the profile of EHOs [has been raised] since COVID and the increased focus on environmental and public health.

Six of the seven interviewees referred to the role of the state government, noting that they (councils) are waiting for state government directives (and funding) to address climate change-related health issues. 

## 4. Discussion

The findings from this study suggest that EHOs are perceived by executives to spend most of their time delivering statutory services relating to inspections and investigations, and related health promotion activities. The inspections and investigation activities conducted by EHOs are driven by the health protection obligations imposed by legislation on councils as outlined in Table 1. 

Executives suggested that broader planning processes accounted for a smaller number of EHO activities. It is also noted that if these activities are conducted, they are likely to be associated with land management, public health, and wellbeing planning activities, with quite limited involvement in climate adaptation and mitigation planning. Interestingly, other departments within local governments were undertaking climate change planning, and if EHOs were involved, it was with public health response activities associated with disaster or emergency management, and not with the adaptation or, from the public health perspective, prevention activities relating to climate change impacts. As such, the focus of adaptation strategies tends to be with impacts on the local built environment and infrastructure. The survey results show that EHOs do participate more in public health and wellbeing planning. In terms of the broader population, and particularly those people seen to be vulnerable to climate change impacts, this would provide both the opportunity and the mechanism to integrate public health climate adaptation and mitigation strategies with other public health and wellbeing strategies. 

The current focus on environmental and infrastructure adaptation strategies does raise questions as to whether council executives are aware of the public health impacts of climate change on their communities outside of the public health emergency management responses required for floods, fires, and extreme weather events, such as heatwaves. These include impacts on:

1. mental health;
2. vector-borne disease and/or zoonoses;
3. food quality and safety;
4. water quality and safety;
5. air pollution and aeroallergens;
6. food availability [30,31,32].

Importantly, it has been recognized that these impacts may be more serious for people who are more vulnerable, including:

1. people who are socio-economically disadvantaged;
2. rural and geographically isolated communities;
3. people with disabilities;
4. children and older people;
5. pregnant women and unborn children;
6. Aboriginal and Torres Strait Islander people [32].

The effect of these impacts and their associated risk factors on the statutory health protection functions, including inspection and investigation activities, is outlined in Table 2.

Table 2 demonstrates that inspections and investigations will be impacted significantly by climate change. Although the health protection objectives remain the same, the scope and complexity of the activities will need to reflect regularly changing public health risk factors. These, in turn, will be dependent on specific localities and their environmental conditions. For example, in times of water shortage and restrictions, a rural food business may need to have frequent changes of drinking water sources from tank, bore, or stream, and possible reuse of wastewater. The public health and business risk elements would have to expand to focus on:

1. the source of water that can be used for commercial food preparation and premises sanitation;
2. the required volumes needed to meet sanitation and production requirements;
3. the required water treatments;
4. time and costs for treatments and analyses;
5. loss of income due to delays and meeting regulatory requirements;
6. overall viability of the business and its value to the local community.

These considerations would then inform the regulatory approach needed to minimize public health risks, safeguard the community, and assist regulated businesses to adjust to climate change impacts. Statutory activities will need to adjust and focus on preventing and moderating the increased risk and resulting harm from local climate change public health impacts, in other words, adapt. Although legislation may set the desired objectives for statutory services, it is the EHOs, through their practice as public and environmental health practitioners, who have the capability of developing and implementing adaptation strategies that best meet local priorities and requirements based on their training in environmental health sciences and local knowledge. 

**Figure 3:**
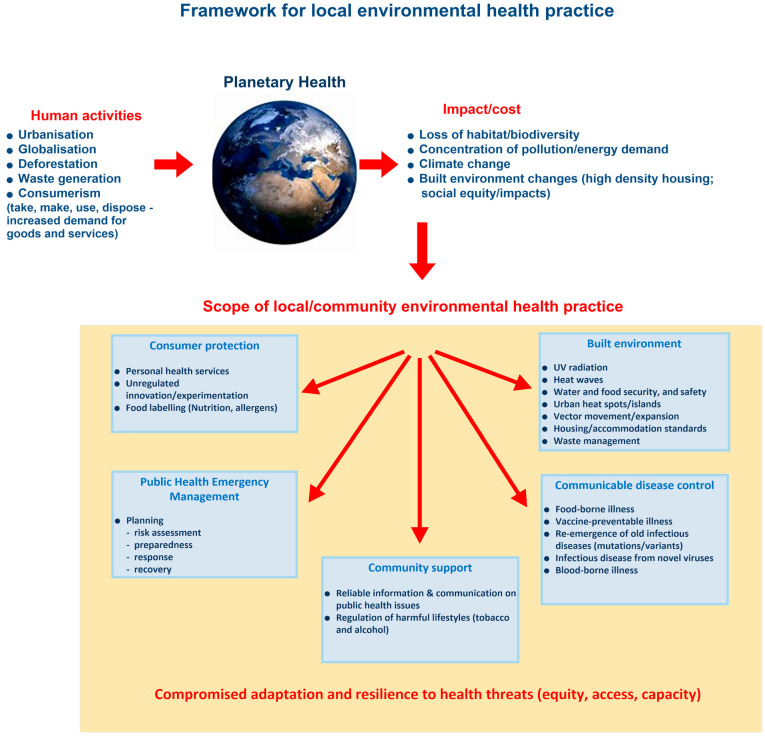
Framework of local environmental health practice. to assist the reconceptualization of the scope of practice required for the planning and response to climate change.

Although some statutory services relate to the regulation of specific businesses, most of these services will be required by the broader community, given the emerging anticipated public health impacts, particularly those who are seen to be vulnerable. The implication is that there may also be increased demand for services [30] and an expansion of some services, such as health promotion activities, as part of the adaptation strategies. As such, local environmental health practice cannot be limited to a narrow set of compliance activities set by legislation and must be concerned with creating health-supporting environments [19]. A critical part of this is building councils’ public health adaptive capacity to prepare and respond to the impacts of climate change. Therefore, the scope of environmental health practice at the local, or community, level needs to be reconsidered by council management and practitioners alike, and conceptualized within a global perspective with an understanding of the interaction of economic and environmental dimensions impacting on local public health. Figure 3 presents a developed framework for local environmental health practice.

The framework acknowledges the impact of human activities on the health of the planet, and the consequential impacts and costs on natural and built environments. These impacts and costs then influence the scope and complexity of local environmental health practice, which can be categorized into five practice domains consisting of:

1. Consumer protection—ensuring compliance safeguards and required safety standards are in place for consumers.
2. Public health emergency management—ensuring the appropriate preparation for (including testing of preparations), response to, and recovery from health impacts of emergencies.
3. Community support—assisting consumers and the broader community in health decision making, raising awareness of public health risks, and improving community public health knowledge.
4. Communicable disease control—minimizing the risk of disease transmitted through food, water, people, and vectors.
5. Built environment—reducing human activities’ impact on the local receiving environments, protecting the community from environmental hazards, and enhancing infrastructure to support health.

Importantly, these domains sit within the context of compromised community adaptation and resilience to health threats, which is determined by issues of equity, access, and capacity that pertain to specific local communities.

Limitations of this study include the small sample size. However, given the similarity of the responses it seems clear that our conclusions about the lack of health planning are unlikely to be limited to those councils that were involved in this study.

## 5. Conclusions

The public health impacts of climate change are being experienced in local communities and will continue to increase in severity. It is critical for governments to plan for the impacts of climate change, and this includes building the capacity to support communities to adapt to environmental changes. This is particularly true for local government, as it is the level of government closest to the community and, thus, must respond on a day-to-day basis to the expectations of its communities. Although climate adaptation and mitigation planning are occurring in local government, it does not include the public health impacts on the community, and this is a concerning oversight. The basis for this oversight seems to be a lack of awareness of the health impacts of climate change outside of a disaster or emergency scenario. Thus, health impacts are not a priority in councils’ overall climate change planning and may explain a lack of willingness, or otherwise, to re-prioritize existing mandated requirements on councils. Although some councils may, as was indicated, wait for the state government to fund them, or otherwise provide resources, many of these issues already fall within their own environmental health services and affect the community they serve. The framework for local environmental health practice presented in this study illustrates that EHOs are not only a critical resource in public health service delivery, which will be heavily impacted on and demanded by climate change, but are also an overlooked and untapped capacity for public health adaptation planning. In local governments, other employees (non-EHOs) have taken leading roles in developing and implementing health plans (e.g., health and wellbeing plans, for example). However, EHOs’ skills in areas such as risk assessment, legislative requirements, policy and planning, and implementation mean that their input into health planning for the future, including in response to climate change, is imperative.

It is recognized that councils are in the unenviable position of balancing a range of priorities, including those imposed by state government legislation, with limited resources. Therefore, it is important that those resources are appropriately harnessed, so the best outcomes are achieved for the community. To achieve this in the climate change context requires that:

1. State governments review their public health priorities and revise the legislation to reflect these priorities and remove conflicting and prescriptive legislative obligations.
2. State government climate adaptation planning integrates with and supports local council adaptation planning.
3. Council executive management recognize the potential for EHOs to increase their capacity for climate change adaptation planning in public health and provide the opportunities to do so.
4. EHOs recognize that they are the public health adaptive capacity for climate change, and engage with councils’ climate change planning processes and adapt environmental health services to reflect the level of community risk based on the framework for local environmental health practice
5. Environmental health officers are the qualified public health practitioners in local government, and they are a critical resource in supporting communities to adapt to the public health impacts of climate change.

## Figures and Tables

**Table 1:** Local government statutory health protection functions by the different Australian states (New South Wales (NSW), Queensland (QLD), South Australia (SA), Tasmania (TAS), Victoria (VIC), and Western Australia (WA)). (Adapted from Lin et al. [17]).

Statutory or Regulatory Functions	NSW	QLD	SA	TAS	VIC	WA
Management/provision of water supplies	Yes	Yes	No	Yes	No	No
Management of waste and sanitation (including domestic wastewater and recycling)	Yes	Yes	No	Yes	No	Yes
Food safety regulation (food-borne illness)	Yes	Yes	Yes	Yes	Yes	Yes
Regulation of businesses of public health interest (recreational waters)	Yes	Yes	Yes	Yes	Yes	Yes
Regulation of lodging houses/prescribed accommodation	No	No	No	No	Yes	Yes
Prevention and control of infectious diseases (arboviral diseases)	Yes	Yes	Yes	Yes	Yes	Yes

**Table 2:** Local health protection statutory functions and related climate change impacts and risk factors. (Adapted from [31,32,33,34]).

Statutory Function/ Service	Public Health Impacts from Climate Change	Risk Factors
Management/ provision of water supplies	Water quality and/or safety	Heavy rainfall resulting in sewerage overflows and drinking water contamination Reduced rainfall or drought and increased demand for water Algal blooms and difficulty controlling water safety and quality Use of unsafe supplies (bores, tanks)
Management of waste and sanitation (including domestic wastewater and its recycling)		Increased domestic wastewater recycling Compromised safety standards from increased demand
Food safety regulation (food-borne illness)	Food quality and safety	Heavy rainfall resulting in flooding and sewerage outflows leading to increased likelihood of microbiological and chemical contamination of food crops Reduced rainfall or drought and increased demand for water Pathogen increase and difficulty controlling water quality Use of unsafe supplies (bores, tanks) Algal blooms and difficulty controlling water safety and quality Bioaccumulation of toxins in fish and seafood Changes in temperature and humidity with heightened occurrence and virulence of food-borne pathogens Increased demand and energy use for food chain temperature and humidity control Reliance on locally sourced products/produce that are cheaper and outside regulatory controls Poor handling hygiene and increased risk of contamination
Regulation of businesses of public health interest (recreational waters)	Recreational water quality and/or safety	Reduced rainfall or drought and an increase in the number and extent of algal blooms Heat stress Increased demand for and resulting difficulty in maintaining water quality (spas and pools) Urban heat islands
Regulation of lodging houses/prescribed accommodation	Heat stress	Lack of capacity to provide heat stress relief in buildings Increased energy demand and costs leading to reduced maintenance and hygiene standards
Prevention and control of infectious diseases (arboviral diseases)	Vector-borne disease and/or zoonoses	Temperature and rainfall changes Accelerated growth of viruses in mosquitoes Changing distribution of vector-borne diseases Environmental changes resulting in persistent/recurring standing water

## Data Availability

Not applicable.

## Supplementary Materials

The following supporting information can be downloaded at: https://www.mdpi.com/article/10.3390/ijerph20031750/s1, Figure S1: Online survey questions; Figure S2: Questions used to prompt follow up telephone interviews; Figure S3: Number of responses from executives from different states; Table S1: Participants’ responses to the question ‘What are the main activities of these EHOs? (tick all that apply)’; Table S2: Participants’ responses to the question ‘If EHOs are involved in climate change/mitigation planning, what specific roles to they fulfil?’; Table S3: Participants’ responses to the question ‘If EHOs are not involved in climate change/mitigation planning and the health impacts associated with climate change, why?’.
