# Supplementary material for: Climate Change and Health: Local Government Capacity for Health Protection in Australia
Source: Int J Environ Res Public Health. 2023 Jan 18;20(3):1750. doi: 10.3390/ijerph20031750 (PMC9914245; doi:10.3390/ijerph20031750)
Supplement: Supplementary file 1 [file ijerph-20-01750-s001.zip › ijerph-2083641-supplementary.pdf]

International Journal of
Environmental Research and Public Health

MDPI

Supplementary Materials

Online survey questions

1. Please enter the name of your council/local government area
2. What is your job title?
3. Do you have executive management responsibility for EHOs?
4. What are the main activities of these EHOs? (tick all that apply)

- Inspections/audits/assessments of regulated businesses/premises (food, personal health, residential etc.)
- Investigation of complaints (unsafe food, environmental hygiene/nuisance, noise etc.)
- Inspections of swimming pools/spas
- Inspections of domestic wastewater management systems
- Legionella control
- Health promotion activities
- Immunization
- Land management planning (development)
- Implementing public health (&/or wellbeing) plans
- Implementing environment/biodiversity plans
- Implementing climate adaptation/mitigation plans
- Implementing stormwater management plans
- Developing public health (&/or wellbeing) plans
- Developing environment/biodiversity plans
- Developing climate adaptation/mitigation plans
- Developing environment/biodiversity plans
- Other activities (for example....)

5. If EHOs are involved in climate change/mitigation planning, what specific roles to they fulfil?

- Project management & coordination tasks
- Project Team member
- Plan implementation - responsible for designated tasks
- Plan development - provider of advice/information
- Other - please specify

6. If EHOs are not involved in climate change/mitigation planning and the health impacts associated with climate change, why not? (choose all that apply)

- EHOs not perceived to have the required skills and knowledge
- There are other more important priorities for EHOs
- The opportunity for EHO participation hasn't been recognized
- There are not enough EHOs
- A lack of interest from EHOs to participate
- Other reasons?
- Because other departments are doing it

7. Would you be willing to have a short discussion on your answers? If so, please provide a contact email address below. (Note: this email address will not be held with your answers from above)

Figure S1: Online survey questions

Interview prompt questions

What is your job title?

Do you have executive management responsibility for EHOs? Yes - if so, how many full time staff, and how many part time?

What are the main activities of these EHOs? (tick all that apply, plus a free text box)

If EHOs are involved in climate change/mitigation planning, what specific roles to they fulfil? (tick all that apply, plus a free text box)

If EHOs are not involved in climate change/mitigation planning and the health impacts associated with climate change, why not? (tick all that apply, plus a free text box)

Would you be willing to have a short discussion on your answers? (text box provided for contact details)

Interview questions

"In your response to our survey, you indicated that EHOs were not involved in health impacts in relation to climate change planning. Can I ask whether health concerns are being considered in council's climate change planning?" If so, which departments are taking responsibility?"

Figure S2: Questions used to prompt follow up telephone interviews

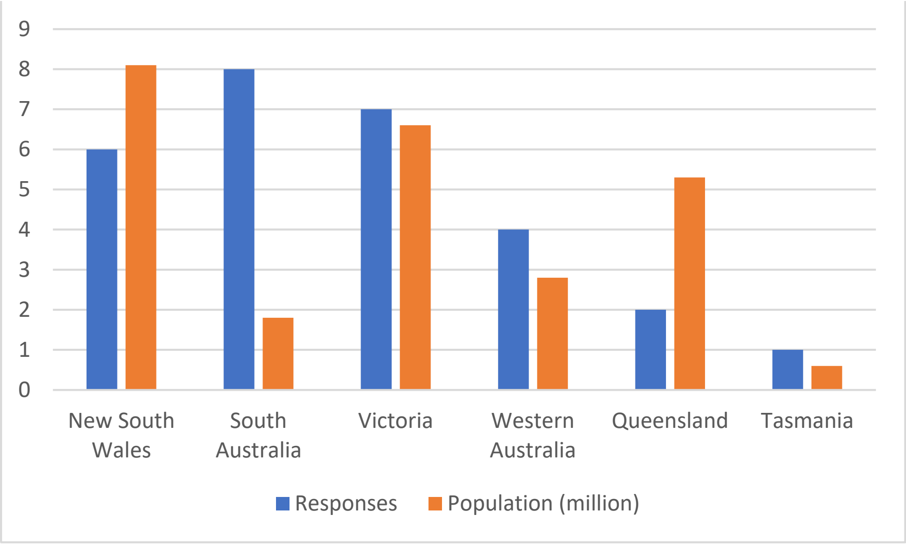

Figure S3: Number of responses from executives from different states

Table S1: Participants' responses to the question 'What are the main activities of these EHOs? (tick all that apply)'

Activity	Count
Inspections/audits/assessments of regulated businesses/premises (food, personal health, residential etc.)	26
Investigation of complaints (unsafe food, environmental hygiene/nuisance, noise etc.)	26
Inspections of swimming pools/spas	22
Inspections of domestic wastewater management systems	18
Legionella control	17
Health promotion activities	19
Immunisation	10
Land management planning (development)	13
Developing public health (&/or wellbeing) plans	11
Developing environment/biodiversity plans	2
Developing stormwater management plans	1
Developing climate adaptation/mitigation plans	2
Implementing public health (&/or wellbeing) plans	13
Implementing environment/biodiversity plans	1
Implementing stormwater management plans	1
Implementing climate adaptation/mitigation plans	1
Other activities (for example....)	3 *

* arborvirus control, asbestos, clandestine laboratories, recreational water monitoring, local laws and temporary events, accommodation parks, footpath dining, liquor licence applications, noise reports and assessment for development approvals and public health complaints - green pools, hoarding, vermin, noise, and pollution.

Table S2. Participants' responses to the question 'If EHOs are involved in climate change/mitigation planning, what specific roles to they fulfil?'.

Role	Count
Project Team member	1
Plan development - provider of advice/information	1
Plan implementation - responsible for designated tasks	0
Other - please specify	2 *

* 1 Specialist advice only. 2. Provision of information to food businesses/health premises etc.

Table S3. Participants' responses to the question 'If EHOs are not involved in climate change/mitigation planning and the health impacts associated with climate change, why?'.

Response	Count
EHOs not perceived to have the required skills and knowledge	5
There are other more important priorities for EHOs	7
The opportunity for EHO participation hasn't been recognised	7
There are not enough EHOs	10
A lack of interest from EHOs to participate	0
Other reasons - stated below	6 *
Because other departments are doing it	18

* 1. No time to develop climate change adaptation/mitigation as a discrete task. It is built into any other relevant task such as the Stormwater Management Plan
2. Currently not a major focus of the city
3. Separate Environmental Services team in Sustainable Assets
4. Lack of understanding of the role they can play
5. We have a Sustainability Officer
6. Climate change plans are managed by other environmental specialists within council.
